# TRPC Channels in Cardiac Plasticity

**DOI:** 10.3390/cells9020454

**Published:** 2020-02-17

**Authors:** Takuro Numaga-Tomita, Motohiro Nishida

**Affiliations:** 1 Department of Molecular Pharmacology, Shinshu University School of Medicine and Health Sciences, Matsumoto 390-8621, Japan; 2 Division of Cardiocirculatory Signaling, National Institute for Physiological Sciences (NIPS), National Institutes of Natural Sciences, Okazaki 444-8787, Japan; 3 Exploratory Research Center on Life and Living Systems (ExCELLS), National Institutes of Natural Sciences, Okazaki 444-8787, Japan; 4 Graduate School of Pharmaceutical Sciences, Kyushu University, Fukuoka 812-8582, Japan

**Keywords:** TRPC channel, cardiac plasticity, calcium signaling

## Abstract

The heart flexibly changes its structure in response to changing environments and oxygen/nutrition demands of the body. Increased and decreased mechanical loading induces hypertrophy and atrophy of cardiomyocytes, respectively. In physiological conditions, these structural changes of the heart are reversible. However, chronic stresses such as hypertension or cancer cachexia cause irreversible remodeling of the heart, leading to heart failure. Accumulating evidence indicates that calcium dyshomeostasis and aberrant reactive oxygen species production cause pathological heart remodeling. Canonical transient receptor potential (TRPC) is a nonselective cation channel subfamily whose multimodal activation or modulation of channel activity play important roles in a plethora of cellular physiology. Roles of TRPC channels in cardiac physiology have been reported in pathological cardiac remodeling. In this review, we summarize recent findings regarding the importance of TRPC channels in flexible cardiac remodeling (i.e., cardiac plasticity) in response to environmental stresses and discuss questions that should be addressed in the near future.

## 1. Introduction

The heart is a pump, delivering oxygen and nutrients to all tissues in the body through blood vessels. During development, heart size and contractile force gradually increase, which is accompanied by structural changes, e.g., hypertrophy, T-tubule formation, and arrangement of sarcomeres [1]. However, the completely developed adult heart changes its structure in response to the hemodynamic load caused by body demands. Flexible changes of heart structure are caused by individual cardiomyocytes and non-cardiomyocyte cells in the heart. Structural changes of cardiomyocytes are directly linked to their contractile force [2]. Therefore, increased oxygen or nutrition demands by the body increase muscle mass of the heart, which is provided by cardiac hypertrophy. In contrast, reduced hemodynamic load to the heart induces a reduction of cardiac muscle mass, which is caused by cardiomyocyte atrophy. In addition, nonmyocyte cells such as cardiac fibroblasts also play important roles to regulate structural integrity and electrical conduction in the heart by regulating extracellular matrix proteins [3]. Although flexible changes of the heart structure are an inherently adaptive feature, many cardiac diseases are brought about by maladaptive cardiac responses to chronic stresses. Therefore, understanding of the underlying mechanism of cardiac plasticity is important for both the prevention of and protection from cardiovascular diseases. 

## 2. Canonical Transient Receptor Potential (TRPC) Channels

The *trp* gene was first discovered in a phototransduction mutant of *Drosophila* [4]. A spontaneous mutation in *trp* resulted in a transient receptor potential in response to continuous light. There are 28 mammalian TRP homologues, which can be further divided into six subfamilies based on genetic and functional similarities: TRPC (canonical), TRPV (vanilloid), TRPM (melastatin), TRPP (polycystin), TRPML (mucolipin), and TRPA (ankyrin). TRP channels have common structural features, including six transmembrane domains, a pore domain between the fifth and sixth transmembrane domains, and a preserved 25-amino-acid sequence termed the ‘TRP domain’. TRPC family proteins, comprising seven mammalian homologues (TRPC1–TRPC7), are the most evolutionally preserved TRP channels with regard to the original form identified in *Drosophila*. Therefore, it was believed and later demonstrated that they function as receptor-activated calcium channels [5]. Among them, TRPC4 and TRPC5 have about 65% amino acid homology, while TRPC3, TRPC6, and TRPC7 have about 75% homology [6]. TRPC1 was originally reported as a candidate subunit of store-operated Ca^2+^ channels [7,8,9,10]. The functional importance of TRPC1 channels is manifested by the promotion of functional coupling between endoplasmic reticulum and the plasma membrane during receptor-induced Ca^2+^ signaling [11]. In addition, it has been shown that mechanical stretch activates TRPC1 in mammalian cells [12]. Thus, TRPC proteins have two important roles: one as a functional channel activated by mechanical stretch or endoplasmic reticulum store depletion and the other as a platform to organize receptor-activated Ca^2+^ channels and intracellular signaling molecules for efficient signal transduction [13].

TRPC channels are widely recognized to be activated downstream of phospholipase C (PLC)-coupled receptors such as G protein-coupled receptors (GPCRs) and receptor tyrosine kinases [13]. As a result of their universal activation mechanism in many cell types, TRPC channels play important roles in basic cellular responses including proliferation, differentiation, and death in response to various environmental stimuli. Recent findings indicate that in addition to PLC-mediated activation, TRPC channels are multimodally activated by environmental stimuli such as mechanical stretch, hypoxia, and oxidative stress [14,15,16,17,18,19]. In particular, TRPC1 and TRPC6 are suggested to be activated by mechanical stimuli [12,20]. Later studies suggested that mechanical activation of TRPC1 and TRPC6 is mediated by phospholipase-dependent mediators [21,22,23,24]. Physiological or pathophysiological conditions expose the heart to changes in mechanical loading and oxygen supply, as well as oxidative stress, especially in the pathological heart. Therefore, TRPC channels play important roles in transducing these environmental stimuli into Ca^2+^ and/or chemical signals within cardiomyocytes. 

All TRPC channels are expressed in cardiomyocytes, except TRPC2. The expression of some of these channels is not high in normal conditions but is increased in the failing heart [25]. According to studies using heterologous expression systems, the TRPC family can be further subdivided into two groups: TRPC1/C4/C5 and TRPC3/C6/C7. Apart from several observations, TRPC channels form functional channels by forming homo- or heterotetramers within these groups. So far, heterologous expression systems have demonstrated that the difference between these two groups involves activation by the PLC product of diacylglycerol (DAG) [26]. TRPC3/C6/C7 is activated directly by DAG, whereas TRPC1/C4/C5 is not [27]. However, Storch et al. recently revealed that DAG activates TRPC4/TRPC5 [28]. Structural analysis with cryogenic electron microscopy revealed the structural conservation of TRPC family channels [29,30,31,32]. This is further supported by the fact that a glycine residue in the selective filter of TRPC3 is both critical to the recognition of lipid mediators and conserved among TRPC channels [33]. Although the actual mechanism of TRPC channel downstream surface receptor activation is still not completely understood [34], its importance in cardiac plasticity has been extensively documented in the model of neurohumoral-factor-induced cardiac remodeling. The original concept of TRPC channels in cardiac remodeling is quite simple. First, major pro-hypertrophic factors such as angiotensin II (AngII) and endothelin-1 activate their receptors, which couple to PLC and downstream TRPC channels. Second, TRPC channels mediate Ca^2+^ influx, which activates major hypertrophic signaling of calcineurin (CaN)/nuclear factor of activated T cells (NFAT). However, recent findings indicate that the involvement of TRPC channels is more complex and chaotic. At present, it is not easy to understand and conceptualize the importance of TRPC channels in cardiac plasticity. Therefore, we herein review recent findings about TRPC channels in cardiac remodeling and raise models and questions that need to be addressed in the near future.

## 3. Functional TRPC Channel Expression and Interaction with Other Proteins

Experiments using heterologous expression have demonstrated that TRPC channels form homo- or heterotetramers to form functional channels in the plasma membrane. Generally, TRPC channels can be subdivided into two groups: TRPC1/C4/C5 and TRPC3/C6/C7. The formation of homo- or heterotetramers is required for the expression of functional channel proteins on the cell surface [35]. However, as these characteristics are demonstrated in heterologous expression systems most of the time, the stoichiometry of functional TRPC channels in an endogenous environment remains elusive. Nevertheless, several lines of evidence indicate the importance of heteromultimerization in native environments. A study of a dominant-negative mutant reported the formation of heterotetrameric TRPC channels during cardiac hypertrophy [36]. We also reported that TRPC6 functions as a negative regulator of TRPC3 coupling to NADPH oxidase 2 (Nox2) through a competitive interaction [37]. 

Another important factor influencing the function of TRPC channels in cardiac plasticity is their subcellular localization in cardiomyocytes. TRPC1 reportedly localizes in cardiomyocytes at sarcolemma and transverse tubules (T-tubules) [23,38]. We and others demonstrated that TRPC3 also localizes to both sarcolemma and T-tubules [36,38,39,40], as does TRPC6 [38]. TRPC4 mainly localizes at sarcolemma, and less at the T-tubules [36,38]. Taken together, TRPC1/C4/C5 are mainly localized in sarcolemma, while TRPC3/C6 are localized in both sarcolemma and T-tubules. As Nox2 is reportedly localized in T-tubules, TRPC3 likely interacts with Nox2 on T-tubules. Strikingly, Doleschal et al. demonstrated that direct activation of TRPC3 or AngII treatment changes the localization of TRPC3 to mainly sarcolemma [40], suggesting that TRPC3 localization is altered in stressed cardiomyocytes.

TRPC channels were originally thought to be the molecular entities of store-operated calcium entry (SOCE) [41]. However, the identification of STIM1 and Orai1 changed this view of the involvement of TRPC channels downstream of PLC activation [42,43,44,45,46]. Several reports have shown that increased STIM1 expression plays critical roles in cardiac hypertrophy [47,48,49,50,51,52]. However, there are complex interactions between the STIM1/Orai1 system and TRPC1/C4 channels that also play important roles in cardiac plasticity [47,53,54,55]. Ohba et al. first demonstrated that the suppression of thapsigargin-induced SOCE was inhibited by silencing of TRPC1 in cardiomyocytes [56]. Sabourin et al. later demonstrated that dominant-negative mutants of TRPC1, TRPC4, and Orai1, as well as siRNA-mediated TRPC5 downregulation, inhibit SOCE in both normal and aldosterone-treated cardiomyocytes [57]. TRPC3 was shown to be activated by Ca^2+^ store depletion in nicotine-treated cardiomyocytes [58]. Yet, how TRPC channels are activated after Ca^2+^ store depletion remains unknown [59]. There must be a functional and/or physical interaction between the STIM1–Orai1 complex and TRPC channels in SOCE in cardiomyocytes, as well as other cell systems [57,60,61]. However, direct activation of TRPC channels by store-depletion or STIM1 is less likely [62]. TRPC might be activated secondary to the Ca^2+^ increase induced by the STIM1–Orai1 complex and subsequent Ca^2+^-dependent PLC activation [63,64]. 

In cardiomyocytes, TRPC channels exhibit functional and physical coupling with other Ca^2+^-mobilizing proteins. All TRPC channels expressed in cardiomyocytes form a complex with the L-type voltage-dependent Ca^2+^ channel (LTCC) Ca_V_1.2 in the embryonic chick heart [65]. Although the precise function of this macromolecular complex remains elusive, increased expression of TRPC channels and fetal genes in the hypertrophied heart highlight the functional importance of TRPC channels in immature cardiomyocytes. Another important interaction partner for TRPC channels is the sodium–calcium exchanger (NCX). TRPC3 reportedly interacts with NCX1 in cardiomyocytes [40,66,67]. Functional coupling between TRPC and NCX has also been recognized in other cellular systems in which Na^+^ entry mediated by TRPC3 drives a reverse mode of NCX that evokes further Ca^2+^ entry into the cell [68]. However, the interaction between TRPC3 and NCX1 plays an important role in the conduction system, rather than structural remodeling. Recently, functional and physical interaction of TRPC3 with phosphodiesterase 1C has been reported. TRPC3-mediated Ca^2+^ entry induces phosphodiesterase 1C activation via Ca^2+^/calmodulin, leading to a reduction of cAMP produced by the adenosine A2 receptor–G_αs_ signaling axis and suppression of the protective effect of adenosine [69]. Downregulation of FKBP52, which has been revealed as a critical interaction molecule for TRPC3, promotes TRPC3-mediated cardiac hypertrophy [70]. Therefore, there may be more complex interplay between TRPC channels and other cation channels in cardiac plasticity. 

## 4. Cardiac Hypertrophy

The heart changes its size and contractility in response to increased or decreased hemodynamic load caused by body demand for oxygen and nutrition supplies [1,2]. The best-known adaptive change of the heart is hypertrophy. However, continued increase of the pressure for hypertrophy leads to maladaptive changes of the heart, ultimately resulting in heart failure. Numerous signaling pathways have been implicated in cardiac hypertrophy [71]. Cardiac hypertrophy is an inherently adaptive response. Athletes or pregnant women experience cardiac hypertrophy, but most of this is completely reversible. The heart size returns to a normal level by attenuation of physical activity or after delivery. Several studies have demonstrated the difference between physiological and pathological cardiac hypertrophy [71]. Physiological and pathological hypertrophy have distinct features at structural, metabolic, and molecular levels. Physiological hypertrophy is accompanied by angiogenesis. In contrast, pathological hypertrophy is accompanied by interstitial fibrosis and reduced capillary densities, which result in myocardial ischemia and lead to heart failure [72,73]. During cardiac hypertrophy, the heart remodels its metabolic pathways to increase ATP production. In physiological hypertrophy, fatty acid and glucose oxidation are increased, in contrast to decreases observed during pathological hypertrophy [74]. In response, the glycolytic pathway is upregulated in pathological hypertrophy, likely to compensate for reduced mitochondrial ATP production. Interestingly, the fetal heart depends on the glycolytic pathway because of the poor availability of fatty acids [71]. Moreover, it has been well recognized that the molecular features of pathological hypertrophy include increased fetal gene expression [75]. Thus, pathological hypertrophy occurs concurrently with a regression to a fetal heart phenotype with regard to metabolic and genetic aspects. 

The best-known signaling pathway regulating physiological hypertrophy is the insulin-like growth factor 1-PI3K(p110α)-Akt pathway. Insulin-like growth factor 1 is important for postnatal development and in the exercised adult heart [76]. Two major pathways have been implicated in pathological cardiac hypertrophy; one is neurohumoral-factor-dependent while the other is biomechanical-stress-dependent [1]. The neurohumoral-factor-dependent pathway is mediated by Gq-type GPCRs, such as AngII receptor type 1 (AT-1) and endothelin receptor. Many signaling pathways participate in pathological hypertrophy, such as small G proteins, mitogen-activated protein kinases, histone deacetylases, and calcium signaling, which are integrated under transcriptional control by several transcription factors. [71,77]. Altered Ca^2+^ signaling, one trigger for cardiac hypertrophy, is likely attributable to changes of PLC and phosphatidylinositol-turnover-dependent increases in intracellular Ca^2+^ [53,71,78]. Although how cardiomyocytes sense and transduce biomechanical signals into hypertrophy is still largely unknown, some reports have demonstrated increased autocrine and paracrine secretion of pro-hypertrophic neurohumoral factors and growth factors in mechanically stressed cardiomyocytes [79]. Alternatively, ligand-independent activation of AT-1 receptors by mechanical stretch might mediate abnormal Ca^2+^ increases [80]. Several Ca^2+^ signaling molecules have been identified as critical regulators of cardiac hypertrophy. Most notably, activity of the Ca^2+^-dependent transcriptional pathway of CaN/NFAT is important for pathological cardiac hypertrophy [78]. Therefore, it is very likely that Ca^2+^-permeable channels play important roles in abnormal Ca^2+^ signaling in pathological cardiac hypertrophy. As a candidate molecular entity for those Ca^2+^ channels, TRPC channels have been intensively studied. 

Both mRNA and protein expression levels of TRPC1 are increased in the hearts of cardiac hypertrophy model rats [55,56,81]. In addition, several experiments demonstrated that TRPC1 plays a critical role in neurohumoral-factor-induced cardiac hypertrophy in vitro [81,82]. TRPC1-knockout mice showed no cardiac hypertrophy after transverse aortic constriction [23]. In addition to these animal model studies, patients with cardiac hypertrophy or heart failure showed increased expression of TRPC1 mRNA [83]. In addition, the involvement of TRPC1 in cardiac hypertrophy was demonstrated in human embryonic stem cell derived cardiomyocytes [83]. As hypertrophied cardiomyocytes have larger SOCE, upregulation of TRPC1 contributes to increased responses that subsequently activate the CaN/NFAT pathway, leading to pathological hypertrophy [55,56]. In addition to this well-known pathway, RNA sequencing of human cardiomyocytes indicates that downstream signaling of TRPC1 involves the NF-κB pathway [83]. 

**Figure 1:**
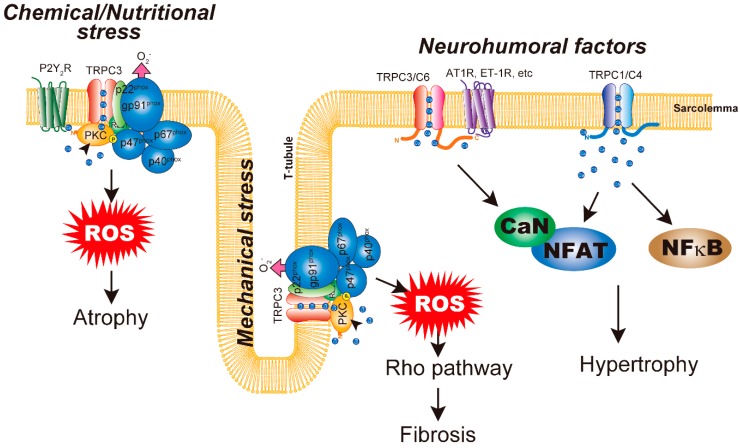
Schematic representation of canonical transient receptor potential (TRPC) channels in cardiac plasticity. Neurohumoral-factor-induced cardiac hypertrophy is mediated by the CaN/NFAT pathway. TRPC3/C6 channels contribute to Ca^2+^ increase downstream of GPCR activation. TRPC1/C4 channels function to regulate background Ca^2+^ entry and the basal level of CaN/NFAT activation in cardiomyocytes. Homomeric TRPC3 channels are upregulated by mechanical stresses. TRPC3 prevents Nox2 from undergoing proteasomal degradation, leading to increased Nox2 protein abundance. TRPC3 is also important for enzymatic activation of Nox2. TRPC3–Nox2 coupling mediates chemical (DOX) and nutritional stress-induced cardiac atrophy. Chemical/nutritional stresses evoke ATP release from cardiomyocytes, which activates G_q_-coupled P2Y_2_ receptors.

Suppression of TRPC4 activity by expression of a dominant-negative mutant of TRPC4 suppressed transverse aortic constriction induced cardiac hypertrophy [36]. Furthermore, the heart expresses several TRPC4 splice variants from α to γ [84,85]. Among these variants, only TRPC4a can interact with PLCβ1b and is expressed on the sarcolemma. Overexpression of TRPC4a in cardiomyocytes induces cardiomyocyte hypertrophy [84]. However, chronic treatment with pro-hypertrophic AngII does not further increase the size of cardiomyocytes. In addition, TRPC4b overexpression has nothing to do with resting or AngII-induced cardiac hypertrophy. In both TRPC4a- and TRPC4b-overexpressing cardiomyocytes, basal activity of the CaN/NFAT pathway is increased [86]. Consistent with these findings, the most important role of TRPC1/C4 seems to be the pathway for background Ca^2+^ entry in cardiomyocytes to fine-tune systolic and diastolic intracellular Ca^2+^ concentration [87]. These results suggest that TRPC4 channel isoforms have different functions in cardiomyocytes and affect downstream targets of universal CaN/NFAT signaling in cardiac hypertrophy (Figure 1). 

Compared with TRPC1 and TRPC4, there are not many reports regarding the function of TRPC5 in the heart. Moreover, reported expression of TRPC5 in the heart and its change during heart failure vary among studies [88,89,90]. Recently we demonstrated that TRPC5 has an anti-hypertrophic effect in cardiomyocytes [91]. Based on the dogma mentioned above, the CaN/NFAT pathway is critical for cardiac hypertrophy. However, purinergic receptor activation by ATP dose not induce hypertrophy, even with a clear increase of CaN/NFAT activity [91]. The reason ATP cannot induce hypertrophy is nitric oxide (NO) production, which is not evoked by other hypertrophic stimulation of AngII or endothelin. TRPC5 activity is linked to endothelial NO synthase activity in endothelial cells [92]. A large body of evidence indicates that NO production likely represents a protective mechanism against cardiac hypertrophy [93]. The suppression of TRPC5 results in a reduction of ATP-induced NO production, thus promoting hypertrophic responses in cardiomyocytes. These results suggest that TRPC channels also function as negative regulators of cardiac remodeling [91]. 

The importance of TRPC3 channels in cardiac hypertrophy was first demonstrated by the Molkentin group, who observed cardiac hypertrophy in cardiomyocyte-specific transgenic mice overexpressing TRPC3 [94]. These mice exhibited elevated SOCE and NFAT activation and cardiomyopathy. This original study clearly indicated increased TRPC3 expression in cardiomyocytes without any specific stimuli causes cardiac hypertrophy. Consistent with these results, a mouse model of left ventricular hypertrophy induced by thoracic aortic banding exhibited increased TRPC3 expression [90]. In addition, infusion of neurohumoral factor also increased TRPC3/C6 expression and induced cardiac hypertrophy [95]. This study clearly indicated that TRPC3/C6 activates the CaN/NFAT pathway and induces pathological cardiac hypertrophy. Nicotine-induced cardiac hypertrophy, which includes NFAT activation, is also suppressed by TRPC3 downregulation [58]. Ectopic expression of TRPC6 in cardiomyocytes has a similar effect as inducing pathological cardiac remodeling [96]. Consistent with the results of studies investigating ectopic expression models of TRPC3/C6, cardiomyocyte-specific overexpression of a dominant-negative TRPC3/C6 mutants suppressed both neurohumoral-factor-induced and pressure-overload-induced cardiac hypertrophy and dysfunction [36]. These gain- and loss-of-function studies of TRPC3/C6 channels prove that they are critical components of cardiac remodeling induced by Gq-coupled receptor-induced cardiac hypertrophy. Recently, we and others reported the suppression of pressure-overload-induced cardiac dysfunction in TRPC3-knockout mice [39,97,98]. Kass’s group reported that TRPC3/C6 double-knockout mice, but not those with single knockout of either channel, became resistant to pressure-overload-induced cardiac hypertrophy [98]. The relationship between TRPC channel activity and the CaN/NFAT activation pathway is widely accepted. However, detailed mechanisms linking TRPC activity to NFAT translocation are still ambiguous. We showed that TRPC3/TRPC6 activation evokes membrane depolarization, which induces LTCC and subsequent NFAT activation [95]. The results of a couple of studies support this hypothesis [99,100], as it was demonstrated that nuclear translocation of NFAT is strongly suppressed by treatment with nifedipine (a selective LTCC antagonist), while persistent LTCC activity alone can evoke hypertrophic responses. Yet, it is likely that Ca^2+^ influx mediated by TRPC channels and coupled NCX partly contribute to NFAT activation.

## 5. Cardiac Atrophy

Cardiac muscle mass is decreased in response to a reduction of hemodynamic load, which is known to be caused by microgravity, long-term bed rest, or cancer cachexia. Cardiac atrophy is mediated by increased protein breakdown by the ubiquitin–proteasome system, Ca^2+^-dependent calpain system, lysosomal system, and autophagy [101]. Hemodynamic unloading by heart transplantation has revealed that unloading stresses increase the expression of calpain proteases [102] and E3 ubiquitin ligases MuRF1 and atrogin-1 [103]. Interestingly, Taegtmeyer’s group observed similar induction of a fetal gene program in atrophied hearts as demonstrated in hypertrophied hearts [75,101]. Activation of the fetal gene program seems to be an adaptive response to metabolic changes of the heart. Therefore, atrophic responses of the heart are not a passive reduction of muscle mass but an active remodeling of heart structure and function occurring in response to a change of hemodynamic load. Compared with hypertrophic responses, underlying mechanisms of cardiac atrophy remain largely elusive. Ca^2+^ signaling likely plays important roles in cardiac atrophy, as the calpain system requires increases of intracellular Ca^2+^ [104], and some reports have demonstrated Ca^2+^ dyshomeostasis in unloaded cardiomyocytes [105,106]. The molecular mechanism underlying cardiac atrophy is presumed based on observations of skeletal muscle atrophy. In skeletal muscle, mechanical unloading causes mitochondrial dysfunction and reactive oxygen species (ROS) production.

In contrast to cardiac hypertrophy, the molecular mechanism of cardiac atrophy remains largely unknown. Doxorubicin (DOX) is a highly effective anticancer drug prescribed for a wide range of cancers. However, the dose-dependent cardiotoxic effects of DOX limits its long-term use. Specifically, the cardiotoxic effects of DOX include arrhythmias induced by its acute effect, the development of left ventricular systolic dysfunction (which leads to dilated cardiomyopathy), and congestive heart failure induced by its chronic effect [107,108]. It was demonstrated that DOX initially induces cardiac shrinkage followed by the induction of myocardial apoptosis and interstitial fibrosis at later stages of left ventricular dilated cardiomyopathy [109,110]. In *Nox2*^–/–^ mice, DOX-induced structural remodeling was completely attenuated [111]. DOX treatment has been shown to increase Nox2 expression in the heart [111,112,113]. Strikingly, the reduction of Nox2 expression by treatment with paeoniflorin suppressed DOX-induced cardiomyocyte apoptosis [112]. In our mouse model, DOX treatment induced cardiac atrophy from 2 weeks after treatment. DOX treatment increased Nox2 protein abundance, which was significantly suppressed by the absence of TRPC3. TRPC3-knockout mouse hearts maintained both whole-heart and cross-sectional areas of cardiomyocytes [113]. Therefore, similar to mechanical overloading, TRPC3 can couple with Nox2 in DOX-induced cardiac atrophy. We recently revealed that nutritional deficiency caused by glucose and amino acid starvation and hypoxia induces cardiomyocyte atrophy [114]. Nutritional stresses evoke intracellular ATP release to the extracellular space, as has been demonstrated in cardiomyocytes and other cells [115,116,117]. Extracellular ATP is the actual signal inducing atrophic responses in cardiomyocytes. As mentioned above, it is known that extracellular ATP does not induce cardiac hypoxia, even with a significant increase of the CaN/NFAT pathway. Atrophic responses of cardiomyocytes could be observed with stimulation of around 1 mM extracellular ATP. A submaximal concentration of ATP (e.g., 100 μM) does not induce atrophic responses. However, the dose responses of ATP to cardiomyocyte atrophy correlated well with intracellular ROS production. Indeed, high ATP stimulation does not increase TRPC3 or Nox2 protein abundances but enhances the interaction between them. Atrophic responses caused by both nutritional stresses and high extracellular ATP are sensitive to the specific P2Y_2_ receptor antagonist, indicating that the P2Y_2_ receptor is upstream of TRPC3–Nox2 coupling. Interestingly, DOX-induced atrophic responses were also suppressed by a P2Y_2_ receptor antagonist. These results suggest the critical involvement of intracellular ATP release and subsequent TRPC3 activation in stress-sensing and transduction of cardiomyocytes (Figure 1).

## 6. Cardiac Fibrosis

Irreversible cardiac remodeling associated with heart failure is manifested by cardiac fibrosis during and after cardiac hypertrophy. Fibrosis is caused by the accumulation of excess fibrous connective tissues, which are mainly composed of collagen fibers. The accumulation of collagen fibers causes stiffening and diastolic dysfunction of the heart. Excess collagen is produced by activated cardiac fibroblasts (CFs), which are also known as myofibroblasts. In addition to collagen, myofibroblasts express several contraction markers and secrete inflammatory cytokines. As tissue stiffening and inflammation can induce transdifferentiation of CFs into myofibroblasts, this pathological feed-forward loop of fibroblast activation progresses cardiac dysfunction. In addition to structural effects on the heart, fibrosis affects electric conduction and, therefore, has arrhythmogenic potential.

It has been demonstrated that CFs express TRPC channels at the mRNA level. However, the expression of specific isoforms in cardiac fibroblasts varies among species. Rat CFs express TRPC1, TRPC2, TRPC3, TRPC5, TRPC6, and TRPC7. However, TRPC1, TRPC4, and TRPC6 are expressed in human CFs. Mouse CFs express TRPC1, TRPC3, TRPC4, and TRPC6. With the exception of TRPC3 and TRPC6, the importance of TRPC channels in physiological and pathological roles of fibroblasts is largely unknown.

We recently showed that TRPC3 single deletion was sufficient to suppress cardiac remodeling in response to pressure overload [39,97]. Interestingly, cardiac fibrosis was diminished in TRPC3-deficient mice in response to mechanical stresses without any effect on cardiac hypertrophy. Mechanically stressed heart tissues exhibit increased TRPC3 protein abundance. Elevated TRPC3 expression induces increased Nox2 protein abundance via a physical interaction that protects Nox2 from proteasomal degradation. In addition, after surface membrane translocation, TRPC3-mediated Ca^2+^ influx recruits essential subunits for Nox2 enzymatic activity via protein kinase C activation, thus regulating Nox2 enzymatic activity. Physical and functional coupling of TRPC3 and Nox2 increases ROS production to supraphysiological levels, leading to eventual cardiac fibrosis (Figure 1). Seo et al. demonstrated that TRPC3/TRPC6 double knockout only slightly suppressed cardiac hypertrophy, suggesting that mechanical stresses also induce hypertrophic responses that are not primarily mediated by TRPC3 and TRPC6. In addition, AngII-induced cardiac hypertrophy is mediated by both TRPC3 and TRPC6 [95]. However, Nox2 only functionally couples to TRPC3 [37]. As TRPC6 is able to interact with Nox2 even in the presence of TRPC3, it can function as a negative regulator of TRPC3–Nox2 coupling [37]. TRPC3 expression is increased in aged and hypertensive rat atrial fibroblasts. Moreover, suppression of TRPC3 by Pyr3 attenuated AngII-induced proliferation, migration, and ECM production in those atrial fibroblasts. AngII induced the expression of profibrotic TGFβ1 and downstream Smad2/3 activation, which were both suppressed by Pyr3 treatment [118]. Therefore, TRPC3 is involved in several key signaling mechanisms of cardiac fibrosis. However, recent publication demonstrated that primary CFs isolated from mice lacking all seven TRPC proteins showed no changes in AngII-induced Ca^2+^ increase but those treated with Orai channels suppressed that almost completely [119]. Since Pyr3 has been revealed to suppress not only TRPC3 but also SOCE [120], the suppressive effect of Pyr3 might be attributable to the suppression of Orai channels, not TRPC3. Yet, Camacho Londono et al. only showed the effect of TRPC suppression on global rise of intracellular Ca^2+^ by AngII. Therefore, TRPC channels are still possibly involved in the regulation of fibrosis-related gene expression as the authors suggest [119]. It has also been demonstrated that TRPC3-mediated Ca^2+^ influx plays an important role in arrhythmogenesis [121]. TRPC3 expression is increased in the atria of patients with atrial fibrillation (AF) and in animal models of AF, whereby it likely induces fibroblast proliferation and differentiation [70]. Pyr3, a TRPC3 inhibitor, reduced ECM protein expression and suppressed AF substrate development in a canine AF model [121]. 

TRPC6 is also involved in cardiac fibrosis [122]. We reported that TRPC6-mediated Ca^2+^ influx functions as a negative regulator of cardiac fibrosis. Treatment of neonatal rat ventricular fibroblasts with endothelin activates NFAT, which subsequently suppresses small G protein Rac and ROS production [122]. However, TRPC6 is reportedly involved in TGFβ1-induced CF proliferation in human cardiac fibroblasts, along with TRPC1 [123]. Molekntin’s group demonstrated that TRPC6 is a critical factor for myofibroblast transformation. TGFβ1 treatment increased TRPC6 expression in dermal and cardiac fibroblasts, and enhanced TRPC6 expression was sufficient for myofibroblast transdifferentiation [124]. In human right ventricular fibroblasts, TRPC6 is also involved in TGFβ1-mediated upregulation of α-smooth muscle actin [125]. In a pressure-overload model, TRPC6 deletion almost completely suppressed cardiac fibrosis. This result arose from the suppression of cardiac fibroblast transdifferentiation into myofibroblasts, as there was no effect on ROS production in the heart (in contrast with TRPC3 knockout) [37]. Furthermore, the orally bioavailable TRPC6-specific inhibitor BI 749,327 could inhibit profibrotic gene expression, reduce cardiac fibrosis, and improve heart function [126]. These results indicate that TRPC6 plays important roles in cardiac fibroblast physiology. 

## 7. Conclusions

TRPC channels were originally recognized as a Ca^2+^ entry pathway to activate CaN/NFAT signaling during cardiac hypertrophy and eventual cardiomyopathy. However, activation of the CaN/NFAT pathway cannot explain all mechanisms of cardiac hypertrophy induced by excessive environmental stresses on cardiomyocytes. Although both TRPC1/C4 and TRPC3/C6 are important for the CaN/NFAT pathway, their contributions are clearly different [87]. In addition, recent findings shed light on the participation of TRPC channels in not only classical cardiac hypertrophy but also other forms of structural cardiac remodeling, fibrosis, and atrophy [39,97,113,114]. We have shown that TRPC3 specifically couples to Nox2 to evoke aberrant ROS production, which leads to both cardiac fibrosis and cardiac atrophy. Mechanical stress causes cardiac hypertrophy and fibrosis; TRPC3 or Nox2 knockout only affect cardiac fibrosis. In contrast, AngII-induced cardiac hypertrophy is attenuated by the suppression of TRPC3 and Nox2. These results suggest that any regulatory mechanism by which the heart discriminates the trigger can lead to different cardiac pathology, even with universal signal mediators of Ca^2+^ and ROS. In addition, TRPC channels are known to be activated by ROS [17,18,19]. Therefore, it is plausible that there is a feedforward loop of ROS-mediated TRPC channel activation that aggravates pathological cardiac remodeling. These results indicate that even with similar phenotypic changes, TRPC channels play different roles depending on the context of environmental stresses. Therefore, to comprehensively understand the contribution of TRPC channels to cardiac remodeling, understanding how different roles of each TRPC channel originated is important.
